# Commensalism or Cross-Species Adoption? A Critical Review of Theories of Wolf Domestication

**DOI:** 10.3389/fvets.2021.662370

**Published:** 2021-04-15

**Authors:** James A. Serpell

**Affiliations:** School of Veterinary Medicine, University of Pennsylvania, Philadelphia, PA, United States

**Keywords:** dogs, wolves, domestication, commensalism, pet keeping

## Abstract

The work of archaeozoologists and molecular geneticists suggests that the domestication of the wolf (*Canis lupus*)—the ancestor of the domestic dog (*C. familiaris*)—probably occurred somewhere between 40,000 and 15,000 years ago somewhere on the Eurasian continent, perhaps in more than one location. Wolf domestication was therefore underway many millennia before the origins of agriculture and the domestication of food animals, such as sheep and goats. Currently, there are two predominant “origin stories” concerning the domestication of the wolf. The dominant narrative in recent literature is the *commensal scavenger* hypothesis which posits that wolves essentially domesticated themselves by invading ancient human settlements in search of animal remains and other edible waste discarded by hunter-gatherers. Over time, tolerance by humans gave a selective advantage to the bolder, less fearful wolves, which then diverged from the ancestral population as they adapted to the new scavenging niche. At some point in the process, humans also began to recognize the benefits of living with resident, semi-domestic wolves, either as guards or as hunting partners, thereby cementing the relationship. The alternative account of wolf domestication is very different. Sometimes known as the *pet keeping* or *cross-species adoption* hypothesis, this narrative draws heavily on anthropological observations of pet keeping among recent hunter-gatherers, and postulates that Paleolithic peoples were similarly inclined to capture, adopt and rear infant mammals, such as wolf pups, and that this habitual human nurturing behavior ultimately provided the basis for the evolution of a cooperative social system involving both species. This review critically examines and analyzes these two distinct domestication narratives and explores the underlying and sometimes erroneous assumptions they make about wolves, Pleistocene humans, and the original relationships that existed between the two species. The paper concludes that the commensal scavenger hypothesis is untenable based on what is known about recent and ancient hunter-gatherer societies, and that wolf domestication was predicated on the establishment of cooperative social relations between humans and wolves based on the early socialization of wolf pups.

## Introduction

Prior to the domestication of livestock animals—sheep, goats, pigs, cattle, horses, llamas, camels, poultry, and so on—all humans lived as subsistence hunters and gatherers who obtained their food directly from nature either by hunting, fishing, or foraging. As far as is known, humans lived like this for at least 2 million years (1) until the closing stages of the last major period of glaciation when, relatively suddenly, some communities in various regions of the world began the process of domesticating plants and animals. This relatively abrupt change from hunting and foraging to domestic food production was one of the most transformative episodes in the history of our species, and it is one that raises a variety of interesting questions. Why, for instance, did humans domesticate plants and animals when they did and not earlier or later, and why in only some geographic regions but not in others? Why also did they domesticate only a small subset of the numerous wild animal species that were potentially available to them, and why, given the many possible choices, did the entire process begin with a large and potentially dangerous carnivore, *Canis lupus*, rather than with something less intimidating and more obviously useful?

The purpose of the current review is to focus specifically on the last of these questions by critically examining the different competing accounts of how and why certain groups of late Pleistocene hunter-gatherers domesticated a large, group-living carnivore, the wolf, the ancestor of the domestic dog. The aim is to re-evaluate some of these different narratives to reveal the underlying assumptions they make about the nature of prehistoric humans and their relations with animals and the natural world.

### The Origin(s) of the Dog

Archaeozoologists and paleogeneticists now suggest that the domestic dog was derived originally from Pleistocene wolves sometime between 40,000 and 15,000 years ago during the Last Glacial Maximum (LGM), possibly in more than one region of Europe and/or Asia (2–7). Wolves were therefore the first animals to be domesticated by humans, preceding the domestication of food or livestock species, such as sheep and goats, by a minimum of 4–5 thousand years. This fact alone raises important questions regarding the possible circumstances and motives leading late Pleistocene hunter-gatherers to single out the wolf for this unprecedented role. It is probably safe to assume that wolf domestication was originally unintentional since Paleolithic humans would have had no concept of the possible future benefits that might arise from such a novel association. At the time, wolves would have competed directly with human hunters for access to similar prey species (8), as well as posing a potential danger to any young or isolated humans who strayed too far from the protection of the group. As such, they would hardly seem an obvious candidate for domestication. Early humans may have hunted and killed wolves opportunistically as occasional sources of food or fur. However, they were never likely to have been a major target of subsistence hunting by Paleolithic peoples because apex predators such as wolves are necessarily less common and more dispersed than their prey (9). So why then did humans and wolves embark on such a risky and seemingly unproductive interspecies collaboration?

Unfortunately, because all of this occurred in prehistory, no written, pictorial, or oral records exist regarding what really happened or why. However, this has not deterred numerous authorities from offering speculative theories that purport to explain how and why our predecessors chose to share their lives and limited resources with this unlikely canine partner. Of these, two very different hypotheses currently dominate the debate, and will be the focus of the present review.

## The Commensal Scavenger Hypothesis

The currently dominant account of wolf domestication posits a world in which late Paleolithic or Mesolithic (Epipaleolithic) hunter-gatherers lived in seasonal hunting camps around which substantial quantities of garbage (animal carcasses and remains, human waste, and so on) accumulated. Attracted to this easy source of food, wolves began scrounging around the garbage dumps, first as occasional visitors and eventually, over time, as permanent or semi-permanent commensal scavengers. Early humans, in turn, tolerated these incursions and, as a result, over multiple generations, the wolves would have become gradually bolder and less fearful of people (10). Later, as the commensal association became more established, the humans would begin to notice the side-benefits of living in association with these animals, such as their tendency to alert to approaching danger or their superior powers of tracking and pursuing game. The humans might then have started giving preferential treatment (e.g., food, protection, etc.) to those individuals that demonstrated these useful traits to the greatest degree, thereby unconsciously initiating a process of artificial selection that resulted in gradual genetic divergence and, eventually, full domestication.

Several versions of this theory can be found in the scientific literature on dog domestication, all of which appear to originate from an imaginative fable first published in the opening chapter of Konrad Lorenz's popular book, *Man Meets Dog* (1953). Here, Lorenz depicts nomadic bands of human hunter-gatherers some 50,000 years ago whose successful hunting activities inevitably attracted the attentions of scavenging wild canids who then began to frequent their hunting camps in search of discarded bones, offal, and so on. After a time, the humans began to realize that, while the scavenging canines were a minor nuisance, they also provided a useful shield against larger marauding predators (e.g., saber-toothed tigers) by barking loudly whenever one was prowling in the vicinity. Now, instead of chasing the dogs away, the humans began encouraging them to remain nearby by actively provisioning them. And so, bit by bit, the process of domestication gathered steam:

“Many years have passed, many generations. The jackals^1^ have become tamer and bolder, and now surround the camps of man in larger packs …. Whereas, formerly they remained concealed by day and only ventured abroad by night, now the strongest and cleverest among them have become diurnal and follow the men on their hunting expeditions” [(11), p. 7].

Though rarely cited, Lorenz's account gained early support from several prominent archaeologists and anthropologists, some of whom argued that so-called “pariah” dogs, the ubiquitous canine scavengers of contemporary Asia, represent surviving relics of just such an early association between humans and wild canids (12, 13). More recent versions of the theory, usually attributed to (10), are also based on firsthand observations of so-called “village” or “dump” dogs—i.e., contemporary free-roaming dogs in parts of Africa and Latin America that exist primarily by scavenging from large municipal waste dumps in densely populated urban areas. Though less elaborate and fanciful, these recent versions of the scavenging hypothesis are nevertheless essentially no different in substance from Lorenz's original narrative. For example, an authoritative account published in the prestigious *Proceedings of the National Academy of Sciences* in 2009 states that:

“Wolf domestication was initiated late in the Mesolithic when humans were nomadic hunter-gatherers. Those wolves less afraid of humans scavenged nomadic hunting camps and over time developed utility, initially as guards warning of approaching animals or other nomadic bands and soon thereafter as hunters” (14).

Similarly, an April 2015 edition of *Science Magazine* reported that:

“Most experts now think dogs domesticated themselves. Early humans left piles of discarded carcasses at the edges of their campsites—a veritable feast, the thinking goes, for wolves that dared get close to people. Those wolves survived longer and produced more pups—a process that, generation by generation, yielded ever-bolder animals, until finally a wolf was eating out of a person's hand. Once our ancestors realized the utility of these animals, they initiated a second, more active phase of domestication, breeding early canines to be better hunters, herders, and guardians” (15).

### Challenges to the Commensal Scavenger Hypothesis

#### Ecological Constraints

Though superficially plausible, this account of wolf domestication is not without its challengers. The eminent geographer, Sauer (16), for example, referred to this theory as “an attractive myth,” and more recently, Jung and Pörtl (17) have strongly disputed the notion that late Pleistocene humans in Europe or Asia reliably generated enough carrion or waste to sustain a permanent population of scavenging wolves. Current evidence suggests that the wolf was already domesticated by the end of the Last Glacial Maximum (LGM) about 15 K years ago (kya) when humans still lived in the relatively small and highly dispersed nomadic groups associated with typical hunting and foraging societies (18). Estimates of human population size and density during this late Pleistocene period are necessarily somewhat speculative, but it is clear that northern hunter-gathers typically live at low or very low densities, proportional to the diversity and abundance of the principal mammalian prey species on which they depend (19). Based on habitable land area during the LGM, and data from 219 different extant hunter-gatherer groups worldwide, recent estimates of the total human population in the Old World (i.e., Africa, Europe, Asia, and Australasia combined) during this period range from 2 to 8 million people, living at densities of between 2 and 12 persons per 100 km^2^ (20). Similarly, population estimates for the whole of Europe during the LGM suggest an average of fewer than 30 thousand individuals (21). Even allowing for the fact that these populations may have been more densely clustered in some areas than in others, this would appear to be insufficient to create a viable ecological niche to support a stable population of scavenging wolves; a species that is reported to need up to 6–7 lbs of high protein food per day in order to reproduce successfully (22).

In the past, advocates of the commensal scavenging hypothesis have argued that wolf domestication actually occurred sometime during the Epipaleolithic/Mesolithic 15–8 kya, despite archaeozoological and paleogenetic evidence to the contrary (13, 14). This period, at least in Europe, was associated with the first appearance of larger and more permanent human settlements, particularly in riverine and coastal areas, and correspondingly greater accumulations of trash or “middens,” mostly consisting of the discarded shells of edible molluscs (23). This desire to place wolf domestication much later than the evidence suggests may reflect the difficulty of reconciling the scavenging hypothesis with the realities of Pleistocene (as opposed to Holocene) hunter-gatherer ecology. In short, human trash dumps and middens of the kind found in Epipaleolithic and Neolithic Europe may have provided limited scavenging opportunities for local carnivores, but they are much too recent to have contributed to wolf domestication.

The only possible exceptions to this rule are the substantial accumulations of predominantly mammoth bones and tusks associated with human activities at various sites in Central and Eastern Europe during the LGM between 40 and 24 kya. Some archaeologists have interpreted these sites as the unused and unwanted by-products of successful mammoth hunts which might indeed have provided important, if temporary, sources of edible carrion for foraging wolves. Other experts, however, have argued that these accumulations of bones and tusks were the result of the deliberate collection and storage of potential building material in regions of steppe tundra where wood for shelter construction was in short supply (23).

#### Cultural Constraints

The ecological problem of finding sufficient edible, organic waste to support a population of scavenging wolves would likely have been exacerbated by hunter-gatherer ritual practices associated with the disposal of the unused remains of hunted animals. Although little is known about the hunting beliefs and rituals of Pleistocene hunter-gatherers, their more recent counterparts are notoriously careful to avoid wasting unusable or inedible portions of the animals they kill and are typically scrupulous about how they dispose of these unused remains (24–32). Referring to one Native American group in central Alaska, the anthropologist, Nelson (28), states that, “[O]ne of the pervasive themes in Koyukon ideology is a prohibition against wasting anything from nature. If someone kills an animal and then leaves it unused or neglects to return for its meat, bad luck or illness will come as punishment. Meat should be carefully butchered and cached where it will not spoil or be defiled by scavengers, and it should be used as fully as possible to avoid offending the animal's protective spirit.” The desire to avoid defilement “by scavengers” is particularly apposite in the current context, suggesting that Paleolithic hunters might have been similarly reluctant to leave any animal remains lying around unused that might have attracted, let alone supported, groups of scavenging wolves.

Certainly, millions of free-roaming dogs currently occupy a commensal scavenging niche throughout much of the developing world (33, 34), so clearly the ability to survive on a diet of human garbage and waste has been an important contributor to the domestic dog's evolutionary success as a species (35). However, these large populations of roaming dogs tend to exist now in areas with long histories of cultural development, high or very high human population densities, and poor or nonexistent means of waste management (36). And even in these circumstances, high rates of adult and pup mortality mean that many free-roaming dog communities are barely sustainable without periodic recruitment from the owned dog population (33, 37). This implies that the scavenging niche is a relatively marginal one in which dogs typically struggle to survive and reproduce successfully without additional human provisioning. Overall, this would indicate that the appearance of permanent populations of canine scavengers was a relatively recent development in dog evolution which only became possible after the first emergence of large towns and pre-urban cities during the Neolithic period around 8,000 years ago, at least 7 thousand years after wolves were supposedly domesticated.

#### Safety Constraints

It is also pertinent to ask why Pleistocene humans would have been interested in tolerating or even encouraging wild wolves to frequent their hunting camps. Supporters of the commensal scavenger hypothesis tend to gloss over this question by assigning an essentially passive and disinterested role to the humans involved in the process. For example:

“People create a new niche, the village. Some wolves invade the new niche and gain access to a new food source. Those wolves that can use the new niche are genetically predisposed to show less ‘flight distance’ than those that don't. Those ‘tamer’ wolves gain selective advantage in the new niche over the wilder ones” (10).

This summary account appears to conflate reduced ‘flight distance’ with true tameness when they are really two separate phenomena. A wild animal that tolerates close approach by humans before fleeing has either lost its fear through repeated, non-threatening exposure (i.e., habituation) or for some reason has never developed a fear response in the first place. An animal that has been tamed, in contrast, not only tolerates human contact but actively seeks it out due to the formation of social bonds. The theory of domestication based on commensal scavenging, at least in its early stages, does not involve tamed wolves—wolves that have been socialized with humans—but rather wild wolves that have habituated to the presence humans and which voluntarily approach and frequent human settlements to gain access to food. The recent history of human-wildlife interactions would suggest that such animals would pose a significant danger to humans, especially to the more vulnerable members of the community. As one pro-wolf website aptly advises:

“when wild animals become habituated to people, they may lose their fear of humans, especially if they are fed or if they associate humans with providing food. Like any large predator, wolves are perfectly capable of killing people. No one should ever encourage a wolf or any other wild animal to approach, and hikers and campers should take all necessary precautions to prevent mishaps involving wildlife” (22).

Attacks on humans by wolves and other wild canids are nowadays unusual, but certainly not unheard of (38). Between 1987 and 2000, five separate attacks on humans by four different, healthy, adult wolves occurred in Algonquin Provincial Park in Canada. In each case, the wolves involved had been frequenting recreational campgrounds where they had received handouts from campers for weeks or months beforehand and had lost their fear of humans (39). Similarly, a much-publicized fatal attack by a pair of coyotes (*Canis l. latrans*) on a lone female hiker in Cape Breton Highlands National Park in Nova Scotia, Canada, in 2009 was linked to long-term habituation and provisioning of local coyotes by tourists. On Fraser Island, Australia, some 279 negative, human-dingo interactions were reported to local authorities between 1996 and 2001 of which 40 were classified as major or catastrophic. The single catastrophic incident involved the killing of a 9-year-old boy and severe mauling of his 7-year-old brother by a pair of habituated dingoes (*Canis f. dingo*). Subsequent analyses determined that habituation of wild dingoes through deliberate or inadvertent feeding by tourists was the foundation for the vast majority of these predatory interactions (40).

Such incidents also appear have been more common in the past, and often in the absence of accidental or deliberate provisioning. An historical review of reported fatal wolf attacks on humans in northern Italy between the fifteenth and nineteenth centuries identified some 600 cases of humans being killed by non-rabid wolves, most of the victims being children under the age of twelve (41). Similarly, careful analysis of French historical archives has unearthed records of more than 3,000 fatal wolf attacks, particularly on women and children, between the fifteenth and twentieth centuries, mostly by healthy (non-rabid) wolves (42). In light of these kinds of evidence, it seems highly improbable that Pleistocene hunter-gatherers would have been comfortable with the chronic proximity of habituated and fearless, scavenging wolves, regardless of their early warning potential.

## The Cross-Species Adoption (or Pet Keeping) Hypothesis

The other competing account of wolf domestication involves the deliberate capture, adoption, hand-rearing, and socialization of wild wolf pups by Pleistocene humans. The original version of this theory was first proposed in 1865 by Charles Darwin's half-cousin, Francis Galton, a Victorian polymath best known for his pioneering work in statistics, meteorology, psychometrics, and eugenics. On the topic of animal domestication Galton wrote:

“It is a fact familiar to all travelers, that savages frequently capture young animals of various kinds, and rear them as favorites, and sell or present them as curiosities. Human nature is generally akin: savages may be brutal, but they are not on that account devoid of our taste for taming and caressing young animals; nay, it is not improbable that some races may possess it in a more marked degree than ourselves” (43).

Setting aside his disparaging depiction of indigenous cultures, Galton's point is that people should not make the mistake of assuming that pet keeping is something that is necessarily restricted to more developed, urbanized societies, but that it may actually be widespread and even more popular among those cultures that urbane Victorians regarded as primitive or “savage.” To reinforce this point, he then went on to provide a lengthy catalog of reports from various noted explorers of the period describing cases of indigenous peoples all over the known world catching, taming and caring for young mammals of various kinds, and keeping them as pets. From here, in Galton's view, it was a matter of common sense to infer that similar pet keeping practices must have existed in prehistoric times and would have led to the eventual domestication of those species that naturally possessed certain characteristics of temperament and behavior that predisposed them to domestic life. These traits, he argued, included hardiness, a tendency to seek comfort or safety, usefulness to humans, a willingness to breed in captivity, tractability (“easy to tend”), and what he referred to as “a fondness for man,” an apparent reference to the animal's ability to form social attachments to humans, for otherwise it would, in his words, “fret itself to death, or escape and revert to wildness.”

Thus, individual wild animal pets that failed to express such traits, or which expressed them only to a limited degree, would have tended to either die of neglect, wander off, or be driven away once they matured past the stage of being appealing as objects of nurturance. Conversely, those in which the traits were more developed would have received favored treatment, been more likely to reproduce as a result and, consequently, been more likely to pass on their desirable domestic traits to their descendants. (43)did not argue that these animals needed to be immediately useful in an economic or practical sense in order to be cared for by humans, though he acknowledged that economic utility would have contributed to their ongoing popularity over time. Instead, he focused on the uniquely human penchant for acquiring and nurturing young animals which, in his opinion, was the essential key to unlocking the doors to domestication.

### Objections to the Cross-Species Adoption Hypothesis

#### Geographic Issues

One general objection to the cross-species adoption hypothesis, first expressed by the anthropologist, Downs (13), was that, while pet keeping is extremely widespread among recent hunting and gathering and horticultural peoples, domestication appears to have been very localized, at least in its early stages. If Paleolithic pet keeping gave rise to domestication, why then, he asks, didn't the two phenomena coincide everywhere? One obvious response to this critique was provided by Galton's (43) original idea—later expanded by Diamond (44)—that not all wild species are equally pre-adapted to domestic life to begin with. If this view is correct, then the geographically localized “hearths of domestication” (16) may simply have been those that happened to support the species of animals that were already pre-adapted to this role and therefore easiest to domesticate.

Additionally, the practice of pet keeping may itself have been localized in prehistoric times. Wholesale cross-species adoption seems to be a uniquely human activity that rarely occurs naturally in other mammals outside of captivity [see (9, 45)]. It must therefore have developed as a cultural characteristic at some point in human evolution, perhaps just prior to the first domestication of wolves. Unfortunately, archaeological evidence of ancient pet keeping is understandably scarce, but there are early indications that wolves, dogs and other canids were sometimes the objects of human admiration and affection. For example, in a burial site at Uyun-al-Hammam in Jordan dating from 17 to 14 kya, archaeologists discovered the well-preserved remains of a fox (*Vulpes vulpes*) that had been buried with two humans. The unusual circumstances of this burial led the authors to conclude that, “rather than the fox being treated as a ‘grave good’ it had a special relationship (i.e., companion) to the humans in these graves” (46). Similarly, some of the earliest archaeological remains of confirmed domestic dogs from later Paleolithic and early Neolithic sites in Europe, Asia and North America were also buried deliberately, either in individual graves or together with humans, in a manner suggesting that these animals were held in high regard by whoever buried them (26, 47, 48). One of these animals even displayed evidence of careful nurturing by humans prior to its death. Recent forensic analysis of dental pathology in a juvenile dog buried with its human owners at the 14 kya site of Bonn-Oberkassel in Germany has revealed that it experienced several debilitating episodes of severe disease, consistent with infection with morbillivirus (distemper), for at least 6 weeks before it died (49). Since morbillivirus infection is typically highly lethal in wild and free-roaming canids, the study authors suggest that this individual could not have survived for as long as it did without, “lasting and intensive human care,” and that this provides, “the earliest known evidence for a purely emotion-driven human-dog interaction” (49). Such findings suggest that at least some late Pleistocene hunter-gatherers, like their more recent counterparts, occasionally developed strong emotional attachments for their canine companions independent of the animals' practical utility or lack thereof.

A further possibility is that only a small subset of Pleistocene hunter-gatherers possessed the necessary incentive to domesticate their pet wolves. An illustration of this point is perhaps provided by the traditional relationships that existed between Aborigines and wild dingoes (*C. f. dingo*) in Australia. According to numerous historical and contemporary accounts, Aboriginal groups in Australia habitually captured and tamed dingo pups and kept them as cherished pets. In most cases, however, these tamed wild dogs were poorly provisioned and undernourished, and usually wandered off and reverted to the wild as adults (50, 51). This situation has raised questions among anthropologists as to why the Aborigines never domesticated—or re-domesticated—the dingo. In the context of the current discussion, however, it seems more appropriate to ask what incentive the Aborigines had to take their relationships with dingoes to this level. Young dingoes were clearly appreciated both as pets and as guardians, particularly among Aboriginal women. Occasionally they also participated in hunting expeditions but seem to have been of marginal practical value in this respect. All of these functions, however, could be provided simply by adopting and rearing new dingo pups from the wild. There was no reason for the Aborigines to retain these animals as adults, particularly given their voracious appetite and tendency to steal food from under the noses of their human partners (51).

In other words, the evolutionary transition from pet wolf to domestic proto-dog may have required an environment that could overcome this obstacle to domestication by providing both humans and their pet wolves with long-term access to sufficient caloric resources to allow both species to survive and reproduce while living together in a combined social group. Interestingly, this type of situation may have existed in some parts of Europe and Asia during the LGM. According to recent estimates, the major mammalian prey assemblages that existed across Northern Europe and Asia during the LGM—and on which both wolves and humans subsisted—would have provided human hunter-gatherers with a surplus of animal protein, particularly during the harsh winter months when plant-based calories would be less available. Unlike wolves, humans are only able to digest about 20% of their energy needs from protein, so any excess could have been fed to pet wolves without depriving humans of nutritional resources. Thus, people and their pet wolves would not have been in competition with each other for limited food resources, thereby enabling them to coexist over multiple generations; long enough to give rise to genetically isolated, breeding populations of proto-dogs (8).

#### Wolves Make Terrible Pets

A more specific objection to the pet keeping narrative focuses on the technical feasibility of taming wolves, or at least taming them to the point where they would be safe to cohabit as pets with human families. In all social mammals, including wolves, there is a short “sensitive period” in early development during which the young form their primary social relationships and attachments (52, 53). Among wolf pups reared under natural conditions this developmental window is relatively narrow, from roughly 1 to 3 weeks of age, thereby ensuring that the pups form their primary social attachments with just their littermates, parents, and other immediate family or pack members (54, 55). After 3 weeks of age, social attraction to unfamiliar individuals is rapidly replaced by social avoidance and fear and, by 5–6 weeks, it becomes increasingly difficult for them to establish social bonds with new partners. Adult wolves can also be socialized and tamed, but the process involves many months of isolation and careful habituation, and the effects may not be extended reliably to other unfamiliar humans (55, 56). As a consequence, modern attempts to produce wolves that are reliably willing to accept humans as social partners have necessitated removing pups from their mothers before 3 weeks of age when they are virtually blind and not yet weaned, and then bottle-feeding them until they are able to properly digest solid food. Even then, these animals often retain temperament traits as adults that make them somewhat difficult and demanding companions, at least in a modern context (10, 57). Such facts have led some authorities to conclude that the whole idea of Paleolithic humans capturing and keeping young wolves as pets is a romantic fantasy (10).

Such sweeping conclusions, however, seem unwarranted based on the evidence. For one thing, it is possible, or even probable, that the temperamental characteristics of Pleistocene wolves made them more amenable to domestic life than their recent equivalents. Due to their history of predation on livestock animals, the modern wolves of Europe and Asia are the products of thousands of years of intensive human persecution (58), so it would not be surprising if they have developed heightened wariness and reactivity in the presence of humans. Additionally, the kinds of temperament traits that render tame wolves unsuitable as pets in modern, urban situations—e.g., escaping and roaming, neophobia, fear of strangers, lack of trainability, predation of domestic livestock, and so on—would have been far less disruptive in a Paleolithic hunter-gatherer context where pet wolves would have lived unrestrained in small temporary settlements, miles away from the nearest unfamiliar human being. Furthermore, we know from numerous first-hand accounts that recent hunter-gatherers not only capture and hand-rear wolves and other wild canids successfully, but also breast-feed the pups that are not yet weaned (9, 51, 59–61). And even if breast-feeding is excluded, adequate human socialization of wolf pups may have been achieved in ancient times simply by removing young pups from the den temporarily and handling them intensively on a regular basis. For example, during a trip to the Alaskan interior in the late eighteenth century, the explorer and naturalist, Samuel Hearne, made the following observation of interactions between wolves and local Indians:

“They always burrow underground to bring forth their young, and though it is natural to suppose them very fierce at those time, yet I have frequently seen the Indians go to their dens and take out the young ones and play with them. I never knew a Northern Indian hurt one of them: on the contrary, they always put them carefully into the den again; and I have sometimes seen them paint the faces of the young wolves with vermillion, or red ochre” [(62), p. 803].

From an evolutionary perspective, it is certainly reasonable to question why hunter-gatherers would visit wolf dens just for the pleasure of playing with the pups, or why they would go to the trouble of hand-rearing and sometimes breast-feeding such pups when they were unlikely to derive any immediate practical or economic benefit from doing so. A possible response to this question would be to argue that cross-species adoption, like intraspecies adoption, is simply a by-product of an evolved propensity for alloparenting and cooperative child-care in the human species that sometimes finds outlets in the adoption and care of unrelated—including non-conspecific—infants (63–66). While such parenting “mistakes” could entail minor evolutionary costs depending on the level of care provided, these might not outweigh the immediate psychological rewards of pet keeping, or the potentially greater inclusive fitness costs incurred by being too discriminating about the allocation of alloparental resources (67).

## Discussion

There are a number of key differences between the two accounts of wolf domestication reviewed in this article. First, in the commensal scavenger scenario, wolves essentially domesticate themselves by voluntarily occupying an ecological niche unwittingly provided by humans. Humans are depicted as playing an essentially passive role in the process; simply tolerating adult wolves in their vicinity until they eventually recognize practical uses for them, such as guarding, hunting, or waste management. As indicated above, a number of fundamental ecological, cultural, and safety constraints render this scenario highly unlikely when viewed from the perspective of Ice Age humans, and the theory also drastically underestimates hunter-gatherer knowledge of, and interest in, socializing and caring for animal pets, including large carnivores such as wolves (43, 60).

In contrast, the cross-species adoption story portrays humans as the primary agents of domestication; deliberately removing young wolf pups from the den and hand-rearing them as dependents. Over time, most of these early pets would have grown up and gradually reverted to the wild, but a small minority—those possessing the most socially desirable traits, such as tameability, trainability, and a tendency to affiliate with humans—might have received favored treatment and hence been more likely to have given rise to domestic descendants with similar characteristics. The final transition from wolves as pets to wolves as domestic proto-dogs may also have required a period of access to surplus animal protein in order to overcome competition for food resources between humans and their adult pet wolves.

The primary appeal of this second narrative is that it is consistent with the frequently observed pet keeping behavior of recent and contemporary hunter-gatherers, while also providing a way around two of the main objections to the commensal scavenger hypothesis: namely, the hunter-gatherer aversion to discarding unused animal remains, and the notion that early humans would have tolerated “fearless” but otherwise unsocialized wolves prowling around their settlements. Pet wolves socialized with humans from an early age would likely have been viewed as dependent group members eligible to partake in shared food resources, at least until they reached adulthood and could fend for themselves. And due to their early socialization experience with humans of all ages, they would not have posed the kind of physical danger to young or vulnerable individuals that is evidently associated with adult wolves that have been merely habituated. On the contrary, their primary affinity with their human foster families would have manifested itself in a motivation to actively defend the group from external threats.

The main weakness of this hypothesis is that it is based largely on observations of recent hunter-gatherer societies which may or may not represent good models of the animal-related attitudes and behavior of their Pleistocene equivalents. Pending the discovery of novel archaeological insights, the only way to address this concern is to point to the obvious ecological similarities between recent and ancient hunting and foraging societies, and the remarkable degree of consistency in animal-related attitudes and beliefs that exists among contemporary hunter-gatherer groups from widely separated regions of the world (25, 27, 68, 69).

A further strength of the cross-species adoption idea is its capacity to explain the transition to economically valuable working partnerships between humans and their earliest proto-dogs. With the exception of scavenging, nearly all of the practical uses and functions of domestic dogs that have given them added value throughout human history, including all the many variations on companionship, hunting, herding, protection, and transport, are predicated on the existence of amicable and cooperative social bonds with humans; social bonds that require early and intensive socialization with people (or their livestock) to be effective and enduring. In contrast, the commensal scavenging idea postulates the development of a human-canine relationship based primarily on habituation—the loss of fearful and avoidant responses—which, on its own, would not have provided a satisfactory basis for the kinds of cooperative working relationships that typify post-Paleolithic human-dog interactions.

Additionally, the commensal scavenger idea is specific to opportunistic carnivores and omnivores that can exploit carrion as a food source and it cannot be easily generalized to explain the domestication of most other species domesticated by Epipaleolithic and Neolithic humans, such as sheep, goats, cattle, buffalo, horses, asses, camels, llamas, alpacas, chickens, turkeys, rabbits, guinea pigs, and so on. The cross-species adoption hypothesis, in contrast, provides a universal mechanism for assimilating otherwise wild animals into the human social milieu; an essential first step in the domestication process (70).

Ultimately, the domestication of the wolf was probably a rare and extraordinary product of unusually optimal ecological conditions in some areas of Eurasia that permitted one or more groups of Pleistocene hunter-gatherers and their pet wolves to coexist and coevolve over multiple generations due to a temporary superabundance of animal protein. Once this relationship was firmly cemented, and wolves were living and breeding entirely within the human domain, unconscious selection for dog-like behavioral traits would probably have been very rapid and increasingly irreversible. The unprecedented animal-human relationship that emerged from this process spread rapidly throughout the human population of the world and has since survived and diversified over tens of thousands of years. In the process, it has doubtless affected the cultural and evolutionary trajectory of our own species in fundamental ways. As with every new technology, the acquisition of dogs extended human senses and capabilities in novel ways, and probably contributed more than tangentially to the post-Paleolithic success of our own species.

## Conclusions

The popular hypothesis that the domestication of the dog from the wolf originated from a commensal scavenging relationship between wolves and Pleistocene hunter-gatherers is untenable for several reasons. Human populations during the Last Glacial Maximum were too small, too dispersed, and too nomadic to reliably generate sufficient edible waste to sustain a specialized population of scavenging wolves. Hunter-gatherer ritual prohibitions against discarding or wasting the remains of hunted animals would likely have further limited wild wolves' access to anthropogenic food sources, while the potential dangers posed by habituated but unsocialized wolves would have discouraged Paleolithic hunters from allowing these animals to approach or frequent their settlements in search of food. Instead, the theory that wolf domestication emerged from the common hunter-gatherer practice of adopting young wild animals and keeping them as cherished pets presents a viable alternative route to wolf domestication. Wolf pups adequately socialized and perhaps breast-fed would not have posed a significant threat to the humans with whom they were familiar and, as adopted family members, would have been provisioned and cared for until old enough to fend for themselves. While the majority were doubtless encouraged to revert to the wild as adults, a small minority, especially those displaying the most dog-like and appealing social behavior, might have been retained through to sexual maturity, perhaps aided by ecological conditions favoring reduced competition with humans for food. Once these pets were able to live out their entire lives with their human foster groups and produce surviving offspring with similar affiliative traits, the stage was set for full domestication and unconscious human selection for other advantageous behavioral variants. Thus, pet keeping, a commonplace hunter-gatherer leisure activity probably derived from alloparenting, accidentally gave birth to a biologically unique and unprecedented human-animal relationship which spread rapidly across human cultures throughout the world.

## Author Contributions

The author confirms being the sole contributor of this work and has approved it for publication.

## Conflict of Interest

The author declares that the research was conducted in the absence of any commercial or financial relationships that could be construed as a potential conflict of interest.

## Footnotes

^1^Lorenz believed erroneously that most modern dog breeds descended from the golden jackal (*Canis aureus*) rather than the wolf. Given the jackal's natural scavenging proclivities, this mistaken assumption may have contributed to his account of domestication.
